# Volutrauma and atelectrauma: which is worse?

**DOI:** 10.1186/s13054-018-2199-2

**Published:** 2018-10-25

**Authors:** Luciano Gattinoni, Michael Quintel, John J. Marini

**Affiliations:** 1 Department of Anesthesiology, Emergency and Intensive Care Medicine, University of Göttingen, Robert-Koch-Straße 40, 37075 Göttingen, Germany; 2 Department of Pulmonary and Critical Care Medicine, Regions Hospital and University of Minnesota, Minneapolis/St. Paul, Minnesota USA

The first recognized form of ventilator-induced lung injury (VILI) was named barotrauma, a word that stresses the role of pressure as a causative agent [1]. Following the work of Dreyfuss et al., which called attention to volume instead of pressure [2], volutrauma was recognized as the primary driver of VILI. While airway pressure distributes across the series-linked thoracic cage and lungs in proportions determined by their relative elastances, volume is a unique variable common to both. Any conceptual distinction between volutrauma and barotrauma vanishes if one considers transpulmonary pressure (PL; stress) instead of airway pressure, and strain (i.e., volume change (∆V) relative to resting lung volume (functional residual capacity (FRC)) instead of tidal volume [3]. These two variables are inextricably coupled by a proportionality constant having the units of pressure (specific elastance, or E_spec_), which in man approximates 12–13 cmH_2_O [4]:

$$ Stress=k\bullet Strain $$

or

$$ PL={E}_{spec}\bullet \frac{\Delta  V}{FRC} $$

Several years after the work of Dreyfuss et al., a new putative cause of VILI was described and called ‘atelectrauma’, focusing attention on repetitive opening and closing of unstable lung units [5]. The concept of atelectrauma quickly gained general consensus and thereafter strongly influenced the practice of mechanical ventilation.

## Volutrauma and atelectrauma: the physical basis

Repeated application of an “excess tidal energy load” to the lung parenchyma is required to induce lung damage. We recently introduced the concept of damaging power [6] as a summary variable encompassing the measurable mechanical causes of VILI: pressures, volume, flow, and respiratory rate. Conceptually, both atelectrauma and barotrauma recognize excessive mechanical energy as causal to repeated nonphysiological strain.

## Volutrauma

Volutrauma is usually interpreted to imply lung overdistension. The constraining physical limit of lung structure is achieved at total lung capacity (TLC; two- to threefold the FRC), a volume at which the collagen fibers are maximally elongated. Damage occurs if the energy applied distends the lung unit repeatedly above its TLC-associated strain [7], in large part through “microfractures” of the extracellular matrix, inflammatory signaling, and vascular stresses that lead to tissue wounding and/or inflammatory activation [8].

## Atelectrauma

Stress and strain amplify at interfaces between regions with different elasticity; these junctions act as “stress risers” [9]. For injured lungs, the stress multiplier (which varies with PL) may exceed 2.0 [10]. One consequence of microatelectasis is the generation of these stress risers, which encourage shearing stresses and also amplify the consequences of applied power to the ‘baby lung’. Apart from opening and closing, these too are relevant forms of ‘atelectrauma’.

Another important but often neglected physical amplifier of stress and strain (‘drop-out’) is rather intuitive. Let us assume that a load of 10 kg is sustained by 10 parallel and interconnected elastic cords that suspend it. Each supports 1000 g (stress) and undergoes similar elongation from its resting baseline (strain). If three of the ten cords do not elongate (in analogy to atelectasis), the remaining seven will support ~ 1430 g each (an increase in stress) and will be proportionally more stretched (an increase in strain).

## Anatomical and biological consequences

Insights into the mechanical causation of VILI are revealed when high ventilating stresses induce injury in previous healthy lungs that initially are free of possible damaging cofactors. Results obtained in animal experiments, however, cannot be translated directly to humans due to important species differences regarding structural tolerance. Indeed, the specific lung elastance, i.e., the proportionality constant between stress and strain that helps quantify the tendency for the lungs to recoil and resist deformation, is ~ 12–13 cmH_2_O in man, ~ 6 cmH_2_O in pigs, and ~ 4 cmH_2_O in rats. Therefore, the transpulmonary pressure required in humans to reach TLC is twice that needed in pigs and three times that in rats. Experimental models, however, do allow us to recognize the basic lesions of volutrauma and atelectrauma. To induce volutrauma in healthy animals requires a very high tidal volume (from 20 to 40 mL/kg). When such large tidal volumes are applied with zero end-expiatory pressure (ZEEP), lesions occur primarily in dependent lung regions where atelectasis-associated sites for stress focusing develop during expiration. Therefore, the model of ‘high-volume’ volutrauma at ZEEP is, in reality, a mixture of volutrauma (high tidal volume) and atelectrauma (dependent atelectasis). Advancing damage leads to reduced aerated lung capacity, functionally increased elastance and impaired gas exchange. The presence of positive end-expiratory pressure (PEEP) may dramatically change the overall picture, depending on its level. In recent long-term experiments conducted in initially healthy prone pigs, we found that while minimal PEEP (4 cmH_2_O) prevented most lesions observed at ZEEP, higher levels of PEEP inflicted damage that progressively manifested as septal rupture and alveolar hemorrhage. Interestingly, ventilation at high PEEP did not impair gas exchange in those experiments, while hemodynamics dramatically deteriorated, in large part due to increased pulmonary resistance and right heart failure.

## Clinical scenario

In animal models the different lesions due to mechanical ventilation may be easily associated with ventilator settings. But in patients with acute respiratory distress syndrome (ARDS), VILI must be inferred from further deterioration of the lung damage already present. By default, therefore, and to sharply discriminate between consequences of different ventilation strategies, the outcome measure in trials has been mortality rate. There is no question that volutrauma prevention primarily requires an appropriately low tidal volume. Unfortunately, however, this mandate promotes atelectasis, prevention of which requires higher PEEP levels. Indeed, the very high PEEP levels needed to achieve near complete reversal of atelectasis may lead to end-inspiratory lung volumes that approach the total capacity of the ‘baby lung’. The danger of this strategy is exemplified, in our opinion, by the dismal outcome of one well executed high-frequency ventilation trial where oscillations were applied at airway pressures normally associated with lung volumes close to the TLC [11].

We believe that the current literature provides clues to resolving the dilemma between atelectrauma and volutrauma. Indeed, three major trials in this field compared the strategy of volutrauma prevention (using low PEEP and low tidal volume) [12] with atelectrauma prevention (using high PEEP and low tidal volume) [13, 14]. The results clearly imply that the risk/benefit of those two strategies were indistinguishable when applied over a PEEP range between ~ 7–15 cmH_2_O. However, the results of a recent clinical trial suggest that a higher PEEP level in association with a high volume/pressure recruitment maneuver augmented mortality incidence [15]. Such data imply that under the conditions of that study the induction of stress-amplified volutrauma may have exceeded the benefit of attenuating tidal opening and closure and reducing the tendency for stress focusing (atelectrauma). Considered together, the extensive body of laboratory and clinical experience indicate that measures should be taken to reduce both plateau and driving pressures, avoid PEEP that is ineffective in recruitment, improve mechanical homogeneity with modest PEEP and prone positioning, and reduce the minute ventilation requirement.

In conclusion, the primary stimulus for VILI is nonphysiologic stretch, a process in which volume expansion and stress focusing are interacting variables. In a sense, therefore, volutrauma and atelectrauma are inextricable phenomena when viewed at the micro level. It follows that the ‘fully open lung’ strategy, which often requires PEEP > 20 cmH_2_O, comes with hazards that pose potentially adverse consequences for both lung parenchyma and hemodynamics that place some subgroups of fragile patients at heightened mortality risk.

## Abbreviations

∆V: Volume change
ARDS: Acute respiratory distress syndrome
E_spec_: Specific elastance
FRC: Functional residual capacity
PEEP: Positive end-expiratory pressure
PL: Transpulmonary pressure
TLC: Total lung capacity
VILI: Ventilator-induced lung injury
ZEEP: Zero end-expiratory pressure
